# Half of UK patients with rheumatoid arthritis are prescribed oral glucocorticoid therapy in primary care: a retrospective drug utilisation study

**DOI:** 10.1186/s13075-015-0895-8

**Published:** 2015-12-24

**Authors:** Rachel J. Black, Rebecca M. Joseph, Benjamin Brown, Mohammad Movahedi, Mark Lunt, William G. Dixon

**Affiliations:** Arthritis Research UK Centre for Epidemiology, Centre for Musculoskeletal Research, Institute for Inflammation and Repair, Manchester Academic Health Science Centre, The University of Manchester, Oxford Rd, Manchester, M13 9PT UK; Department of Medicine, The University of Adelaide, Adelaide, SA 5005 Australia; NIHR Manchester Musculoskeletal Biomedical Research Unit, Central Manchester University Hospitals NHS Foundation Trust, Manchester Academic Health Science Centre, 29 Grafton Street, Manchester, M13 9WU UK; Health e-Research Centre, Farr Institute for Health Informatics Research, Manchester Academic Health Science Centre, The University of Manchester, Oxford Rd, Manchester, M13 9PL UK; Department of Rheumatology, The Basil Hetzel Institute for Translational Health Research, Woodville Rd, Woodville South, SA 5011 Australia; Department of Rheumatology, Salford Royal NHS Foundation Trust , Stott Lane, Salford, M6 8HD UK

**Keywords:** Glucocorticoids, Rheumatoid arthritis, Drug utilisation, Primary care

## Abstract

**Background:**

Patients with rheumatoid arthritis (RA) have shared care between rheumatologists and general practitioners (GPs). Rheumatologists guide immunosuppressive therapy, whilst GPs rely on analgesia and glucocorticoid (GC) therapy to manage active disease. The objective of this study was to describe patterns of GC prescribing for patients with RA in primary care and to determine the influence of patient characteristics and prescriber.

**Methods:**

Incident RA patients were identified within the Clinical Practice Research Datalink, a United Kingdom (UK) primary care research database. Descriptive statistics identified patterns of oral GC prescribing. Prescribers were categorised by their tendency to prescribe GCs (high/low). Logistic regression was used to identify baseline characteristics associated with GC prescriptions during follow-up and to examine whether baseline characteristics influenced prescribing differently in high versus low prescribers.

**Results:**

A total of 7777 patients (47 %) received ≥1 GC prescription during follow-up. The average daily dose was 7.5 mg (IQR 5–15.3 mg). Of those who received GCs, >50 % were prescribed >10 mg/day and 20 % >30 mg/day. The median proportion of time spent on GCs was 26.3 % (IQR 3.8–70.0 %). Age and cardiovascular disease (CVD) were associated with increased likelihood of receiving GCs. High prescribers more commonly prescribed GC therapy in older patients and patients with hypertension.

**Conclusions:**

Half of patients with incident RA received GCs in primary care. Average GC use was 7.5 mg for 25 % of the time, perhaps higher usage than rheumatologists and GPs might expect. GCs were prescribed more commonly in certain high-risk populations, including older patients and those with CVD.

## Background

Glucocorticoid (GC) therapy was first introduced as a treatment for rheumatoid arthritis (RA) over 60 years ago [1]. GCs have potent anti-inflammatory properties that rapidly relieve joint pain, swelling and stiffness and also prevent structural damage [2]. However, they are associated with significant and predictable side effects (SEs) of concern to both patients and physicians [3, 4]. In the general population, GCs account for 2.5 % of all adverse drug reactions leading to hospital admission [5]. Guidelines for rheumatologists advocate short-term use of GCs [6], an acknowledgment that longer duration of therapy is associated with increased risk of developing certain SEs such as infection [7].

The management of RA, a condition where disease exacerbations are part of the natural history, involves shared care between the treating rheumatologist and the patient’s general practitioner (GP). Shared care for patients with RA is recommended in many international guidelines and standards of care [8–11] and is common practice in the UK. In early RA, GPs will often commence initial therapy, which may include simple analgesia, non-steroidal anti-inflammatory drugs (NSAIDs) and GCs. The treating rheumatologist then guides ongoing management with advice about disease-modifying anti-rheumatic drugs (DMARDs) and GC use. When faced with active disease, rheumatologists can initiate changes to DMARD therapy, however GPs rarely alter DMARDs and their options for treating disease flares that occur between rheumatology appointments are usually limited to GCs and analgesia. In some instances, GPs will initiate or continue GCs based on the recommendation of the treating rheumatologist, and on other occasions they may initiate therapy based on their own clinical judgement. The extent and pattern of GC prescribing for RA in primary care has not been well quantified or described. Therefore, it is not known if doses and duration of treatment are in keeping with current guidelines. It is important to understand if certain patient groups are more likely to receive GCs in primary care, in particular those at increased risk of developing GC SEs such as older patients or those with pre-existing comorbidities. Doctors are known to have differing beliefs about GC use and its risks [12–14], therefore it is also important to understand if certain doctors prescribe more GCs.

The purpose of this study is to examine how oral GCs are prescribed for patients with RA in UK primary care. The primary objective is to describe overall drug utilisation and patterns of dose and duration. Secondary objectives are to explore the association between patient characteristics and GC use, and to examine variability in prescribing practices between prescribers.

## Method

### Data source

This study utilised the Clinical Practice Research Datalink (CPRD) [15], an automated database that contains pseudonymised, prospectively collected electronic medical records from registered UK general practices. In the UK, healthcare is centralised through GPs and electronic medical records are maintained and updated within general practices. The electronic medical records contain all primary care details plus information about referrals. Anonymised prescriber and practice codes are also available as part of the CPRD dataset.

Studies have found the data held by the CPRD to be representative of the UK population in terms of age and gender structure [16, 17]. Validation studies have demonstrated good completeness and accuracy of the data, particularly for chronic diseases [18, 19]. The CPRD has its own internal quality measures at the patient and practice level, including acceptability flags based on contiguity and quality of patient data, and an ‘up to standard’ date for practices based on continuity of data recording [20]. UK primary care electronic medical records use a unique coding system with Read codes assigned to medical diagnoses and Product codes assigned to medications [21].

### Population and follow-up period

**Fig. 1:**
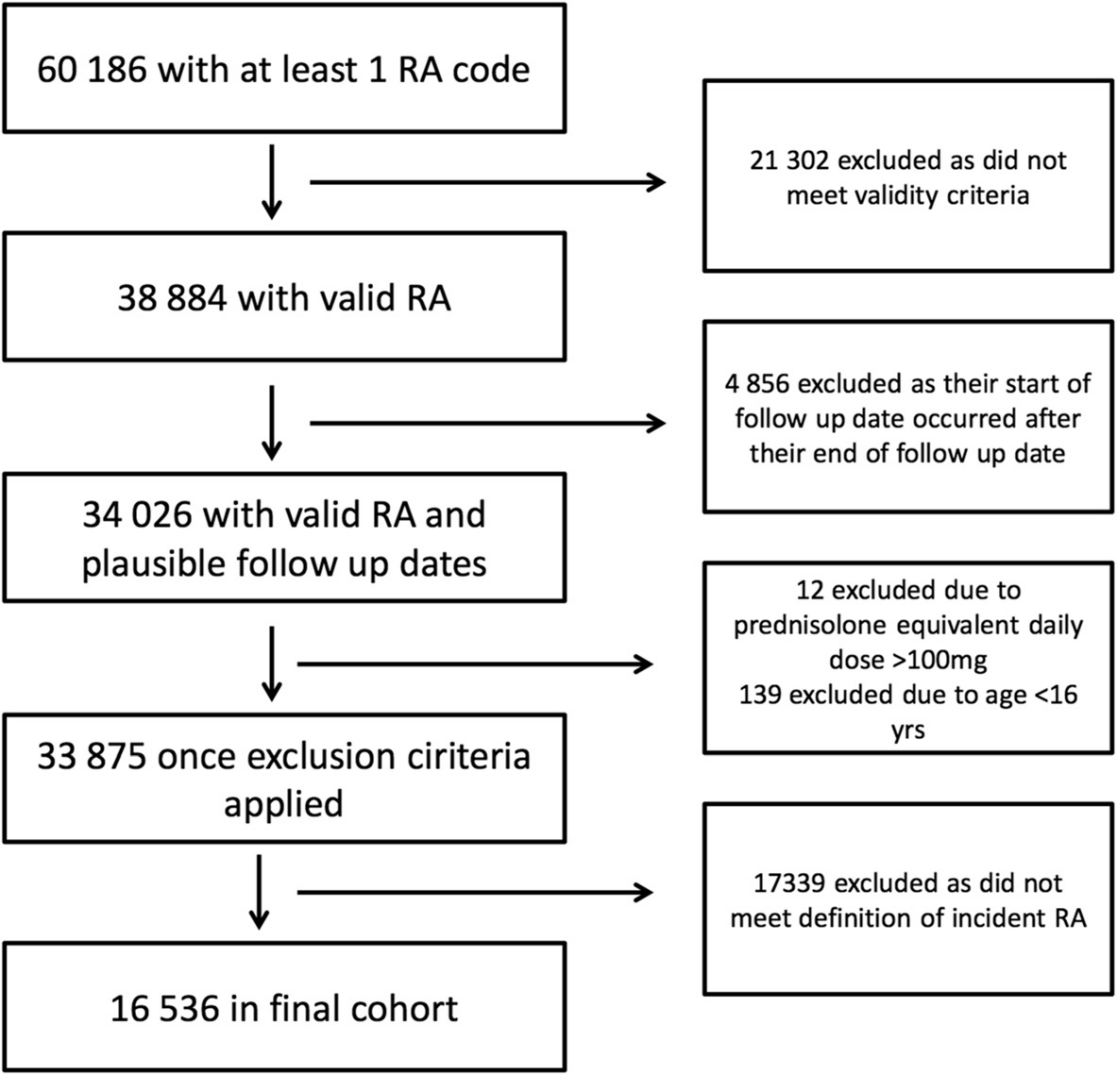
Steps taken to obtain the final cohort

Figure 1 outlines how the cohort of incident RA was derived. All patients with an RA code recorded in CPRD between 1 January 1992 and 31 December 2009 were identified. A validated algorithm, shown to have a sensitivity of 84 % and a specificity of 86 % when compared to the American College of Rheumatology 1987 revised RA classification criteria [22], was then used to identify patients with true RA. The RA diagnosis date was defined as the date of the first RA code in patients with validated RA. Patients with incident RA were identified as those with an RA diagnosis date on or after the 1 January 1992 and at least 12 months of data recorded prior to diagnosis. Those with a GC prescription greater than a pre-defined maximum prednisolone equivalent dose of 100 mg per day and those aged less than 16 years were excluded. Follow-up began on the date of RA diagnosis and ended when the patient left the practice, died, the study period finished (31 December 2009) or on the date data was last collected from the practice, whichever occurred first.

**Table 1:** Patient characteristics thought to be potentially relevant to GC prescribing practices

Patient demographics	Age	
	Gender	
GC-associated comorbidities	Musculoskeletal	Osteoporosis, avascular necrosis, myopathy
	Endocrine/metabolic	Diabetes
	Cardiovascular	Hypertension, hyperlipidaemia, cardiovascular diseases (myocardial infarction, angina, stroke)
	Gastrointestinal	Peptic ulcer disease, pancreatitis
	Psychological/behavioural	Depression, psychosis, insomnia
Inflammatory comorbidities	Respiratory	Chronic obstructive pulmonary disease, asthma, lower respiratory tract infections
	Skin diseases	Atopic eczema, cutaneous vasculitis, cutaneous lupus
	Gastrointestinal diseases	Inflammatory bowel disease (ulcerative colitis, Crohn’s disease)
DMARDs	Methotrexate	
	Sulfasalazine	
	Hydroxychloroquine	
	Leflunomide	
	Other DMARDs	Cyclosporine, azathioprine, penicillamine, chloroquine, gold

*GC* glucocorticoid, *DMARDs* disease-modifying anti-rheumatic drugs

### Outcome measure: glucocorticoid prescriptions

All prescriptions of oral GCs (prednisolone, cortisone, hydrocortisone, triamcinolone, methylprednisolone, dexamethasone, bethamethasone, budesonide and deflazacort) were identified. Dosages were converted to a prednisolone-equivalent daily dose. Patients were classified as having ever or never been prescribed oral GCs according to receipt of at least one prescription during their follow-up period.

### Predictors: patient characteristics and prescriber tendency

Baseline patient characteristics postulated to potentially influence prescribing were divided into demographics, other inflammatory indications for GCs, GC-associated comorbidities (e.g. osteoporosis, diabetes) and DMARD use as a surrogate for disease severity (Table 1). Characteristics and DMARDs were defined as being present at baseline if the diagnosis date or first prescription date occurred on or before RA diagnosis date.

The primary (i.e. most frequent) prescriber of all medications was determined for each patient. For each primary prescriber, the mean proportion of time their patients spent on GCs during follow-up was calculated by dividing the length of time spent on GCs by the length of follow-up time for each patient and then determining the mean of this value amongst all patients seen by a given primary prescriber. Prescriber tendency was then assigned as ‘high’ or ‘low’ prescribers based on whether the mean proportion of time their patients spent on GCs was above or below the median value.

### Statistical analysis

#### Descriptive analysis

Descriptive statistics were used to identify patterns of GC prescribing including: ever use (yes/no), dose, duration and then dose and duration combined. Ever use was determined as a binary yes/no variable for the observation period and separately for the 12 months prior to diagnosis. For patients who received at least one GC prescription during the observation period, the average, lowest and highest prednisolone equivalent daily doses were determined for the time they were exposed. The median of these values across all treated patients was then calculated as a population summary statistic. The proportion of patients to ever receive greater than 5 mg, 10 mg, 20 mg and 30 mg per day was also determined.

The total number of patient years contributing to the follow-up period was determined, as was the number of patient years spent on and off GCs. For each patient that ever received GCs, the duration of follow-up, duration on GCs and proportion of their total follow-up time spent on GCs was determined. The median of these values was calculated as a population summary statistic.

A GC course was defined as consecutive GC prescriptions where the end date of the first prescription was not more than one calendar day different from the start date of the next prescription. The median number of courses per year and the number of patients with a single course longer than 3 months and 1 year was determined. Finally, dose and duration were combined and the proportion of patients taking more than 5 mg/day and 10 mg/day for greater than 3 months and 1 year was calculated.

The number of patients receiving a GC injection and the median number of injections per patient was also determined.

#### Patient characteristics

Univariate logistic regression, adjusted for age and gender, was initially carried out and then stepwise logistic regression was used to identify baseline patient characteristics associated with GC prescriptions (ever versus never) including demographics, other possible inflammatory indications, GC-associated comorbidities and DMARDs.

#### Prescriber tendency

Univariate logistic regression with an interaction term was used to determine the effect of prescriber tendency on the likelihood of receiving a GC prescription (ever versus never) during follow-up and to test the interaction between prescriber tendency and baseline patient characteristics, including patient demographics and GC-associated comorbidities.

All analyses were carried out using Stata version 12.1 (StataCorp LP, College Station, TX, USA). The study was approved by the CPRD Independent Scientific Advisory Committee (protocol 11_113RA2).

## Results

From 1 January 1992 to 31 December 2009, 60,186 patients had at least one code for RA. Once the validated algorithm was applied to this cohort, 38,884 RA patients from 636 general practices across the UK were identified. Of these, 4856 were excluded because their RA diagnosis date occurred after their end of follow-up date, 12 were excluded due to a prednisolone-equivalent daily dose greater than a pre-defined maximum of 100 mg per day and 139 were excluded because they were aged less than 16 years. A total of 16,536 remained in the final cohort after the definition for incident RA was applied as shown in Fig. 1. The majority of the patients were female (69.3 %) and the median age was 59.8 years [interquartile range (IQR) 49.0–70.3].

### Patterns of GC use

#### Ever use (yes/no)

There were 7777 (47 %) patients who received at least one oral GC prescription during the study period and these patients were classified as ever receiving a GC prescription. There were 3412 patients (20.6 %) who received a GC prescription in the 12 months preceding their diagnosis. Of these, 2940 (86.2 %) were prescribed further GCs during the follow-up period.

#### Dose

For those that ever received GCs, the population distribution was a median of 7.5 mg per day (IQR 5–15.3 mg) for the average dose, 5 mg per day (IQR 2.5–7.5 mg) for the lowest dose and 15 mg per day (IQR 7.5–30 mg) for the highest dose. Of those prescribed GCs during follow-up, 83 % ever received a prednisolone equivalent daily dose of more than 5 mg/day, 58 % more than 10 mg/day, 39 % more than 20 mg/day and 18 % more than 30 mg/day.

#### Duration of GC therapy

Total follow-up time was 92,641 patient years (mean 5.6 years/patient), with 14,382 (15.5 %) patient years spent on GCs and 78,259 (84.5 %) patient years spent off GCs. Of the 7777 patients who received GCs during follow-up, the median duration of follow-up time spent on GCs was 0.80 years (IQR 0.15–2.56) and the proportion of time spent on GCs was 26.3 % (IQR 3.8–70.0 %).

Of those who received GCs in the 12 months prior to diagnosis, the median proportion of time spent on GCs during that year was 22.7 % (IQR 5.4–67.2 %). Table 2 summarises GC doses and duration of use.

**Table 2:** Summary statistics of GC doses and duration of use per patient during follow-up and in the 12 months prior to study entry for those patients ever prescribed GCs (n = 7777)

	Follow-up period		12 months prior to study entry	
Measure^*^	Median	IQR	Median	IQR
Duration of follow-up (years)	5.29	2.62–8.58	-	-
Cumulative duration of GC use (years)	0.80	0.15–2.56	0.23	0.05–0.67
Proportion of follow-up time on GCs (%)	26.3	3.8–70.0	22.7	5.4–67.2
Average dose^**^ (mg)	7.5	5–15.3	10	5–20
Lowest dose^**^ (mg)	5	2.5–7.5	5	3–15
Highest dose^**^ (mg)	15	7.5–30	15	6–30

*GC* glucocorticoid, *IQR* interquartile range

^*^Summary statistics were obtained by calculating the value for each patient and then determining median values across the whole population

^**^All doses are prednisolone-equivalent daily doses

Of those that received GCs, the median number of GC courses throughout follow-up was 5 (IQR 2–12) and the median number of courses per year was 1.4 (IQR 0.4–3.0). The number of patients that received more than two GC courses per year was 3060 (39.3 % of those that received GCs). The median duration of each GC course was 50 days (IQR 21–111). Of those that received GCs, 57.1 % received a course longer than 3 months and 13.1 % were prescribed a GC course lasting longer than 1 year.

#### Dose and duration of treatment

Of those prescribed GCs, 26.6 % received continuous treatment with more than 5 mg/day for longer than 3 months and 2.4 % received continuous treatment with greater than 5 mg/day for longer than 1 year. 4.7 % received more than 10 mg/d for more than 3 consecutive months.

#### GC injections

There were 8373 prescriptions for GC injections (intramuscular, intra-articular and periarticular) during the study period in 2911 patients (37 % total cohort). The majority were for methylprednisolone (72 %), followed by triamcinalone (21 %), prednisolone (4 %) and hydrocortisone (3 %). The median number of injections per patient was 2 (IQR 1–3).

### Patient factors associated with GC prescribing

Each 10-year increase in age was associated with a 17 % greater likelihood of being prescribed GCs [odds ratio (OR), 1.17 95 % confidence interval (CI) 1.14–1.20]. Higher GC prescribing was seen in patients with inflammatory comorbidities of the lung: asthma (OR 1.58, 95 % CI 1.42–1.76), chronic obstructive pulmonary disease (OR 1.63, 95 % CI 1.33–1.99) and lower respiratory tract infections (OR 1.22, 95 % CI 1.11–1.34). However, there was no association with inflammatory conditions of the skin or gastrointestinal tract (GI) tract (Table 3).

**Table 3:** Baseline patient characteristics associated with GC prescriptions

Variable	Ever GC use (number, %)	Never GC use (number, %)	Univariate analysis^*^ (odds ratio, 95 % CI)	Multivariate stepwise analysis (odds ratio, 95 % CI)
Baseline demographics				
Age (decades)			1.02, 1.02–1.02^**^	1.17, 1.14–1.20
Gender (female)	5313, 68.32 %	6153, 70.25 %	0.94, 0.88–1.00	
Current smoking (versus never)	2147, 27.61 %	2385, 27.23 %	1.04, 1.00–1.08^**^	1.22, 1.13–1.32
Baseline GC-associated comorbidities				
Osteoporosis	427, 5.49 %	279, 3.19 %	1.42, 1.21–1.66^**^	
Avascular necrosis	7, 0.09 %	4, 0.05 %	1.68, 0.49–5.78	
Myopathy	14, 0.18 %	10, 0.11 %	1.35, 0.60–3.08	
Diabetes mellitus	603, 7.75 %	699, 7.98 %	0.85, 0.76–0.95^**^	0.71, 0.62–0.82
Cardiovascular disease	364, 4.68 %	264, 3.01 %	1.22, 1.04–1.44^**^	1.25, 1.03–1.51
Hypertension	1842, 23.69 %	1754, 20.03 %	0.98, 0.90–1.06	
Hyperlipidaemia	798, 10.26 %	830, 9.48 %	0.93, 0.84–1.04	0.86, 0.76–0.97
Peptic ulcer disease	382, 4.91 %	334, 3.81 %	1.13, 0.97–1.32	
Pancreatitis	46, 0.59 %	43, 0.49 %	1.11, 0.73–1.70	
Depression	1684, 21.65 %	1847, 21.09 %	1.11, 1.03–1.19 ^**^	
Insomnia	985, 12.67 %	865, 9.88 %	1.21, 1.10–1.34^**^	
Psychosis	56, 0.72 %	52, 0.59 %	1.24, 0.84–1.81	
Baseline inflammatory comorbidities				
Chronic obstructive pulmonary disease	540, 6.94 %	189, 2.16 %	2.74, 2.31–3.25^**^	1.63, 1.33–1.99
Asthma	1492, 19.18 %	925, 10.56 %	2.07, 1.89–2.27^**^	1.58, 1.42–1.76
Lower respiratory tract infection	1717, 22.08 %	1342, 15.44 %	1.47, 1.35–1.59^**^	1.22, 1.11–1.34
Inflammatory bowel disease	78, 1.11 %	63, 0.72 %	1.35, 0.96–1.89	
Cutaneous lupus	13, 0.17 %	11, 0.13 %	1.30, 0.58–2.93	
Cutaneous vasculitis	6, 0.08 %	0, 0.00 %	1	
Atopic eczema	1084, 13.94 %	1127, 12.87 %	1.10, 1.00–1.20^**^	
Baseline DMARD use				
Methotrexate	465, 5.98 %	501, 5.72 %	1.07, 0.93–1.22	0.80, 0.66–0.97
Sulfasalazine	468, 6.02 %	581, 6.63 %	0.91, 0.80–1.03	0.69, 0.58–0.83
Hydroxychloroquine	259, 3.33 %	256, 2.92 %	1.20, 1.00–1.43^**^	
Leflunomide	105, 1.35 %	71, 0.81 %	1.83, 1.35–2.49^**^	1.75, 1.18–2.59
Other DMARDs^***^	277, 3.56 %	151, 1.72 %	2.06, 1.68–2.52^**^	1.68, 1.28–2.19

*GC* glucocorticoid, *DMARDs* disease-modifying anti-rheumatic drugs, *CI* confidence interval

^*^Adjusted for age and gender

^**^Significant in univariate analysis

^***^Other DMARDs include gold, penicillamine, cyclosporine, chloroquine and azathioprine

The association with pre-existing comorbidities known to be associated with GC therapy was less consistent. GC prescribing was higher in patients with pre-existing cardiovascular disease (CVD) (OR 1.25, 95 % CI 1.03–1.51) and in current smokers (OR 1.22, 95 % CI 1.13–1.32) but lower in patients with diabetes mellitus (DM) (OR 0.71, 95 % CI 0.62–0.82) and high cholesterol (OR 0.86, 95 % CI 0.76–0.97). There was an association with osteoporosis, depression and insomnia seen in the univariate model, but these factors were not included in the final multivariate model. There was no significant association with other GC-associated comorbidities at baseline including avascular necrosis, myopathy, hypertension (HTN), peptic ulcer disease (PUD) or pancreatitis.

A previous GC prescription prior to RA diagnosis was the strongest predictor of ever receiving a prescription post-diagnosis (OR 9.50, 95 % CI 8.51–10.60). GC prescribing was lower with baseline methotrexate and sulfasalazine use, but higher with leflunomide and ‘other’ DMARD use.

### Prescriber tendency and GC prescribing

In total 3270 GPs were assigned as primary prescribers. The mean proportion of time their patients spent on GCs ranged from 0 to 100 % (median 11.3 %, IQR 0.11–25.9 %). GPs were thus categorised as ‘high’ prescribers if their patients spent a mean of ≥11.3 % of follow-up on GCs. A total of 6427 (38.9 %) patients were assigned a ‘low’ GC prescriber and 10,109 (61.1 %) were assigned a ‘high’ GC prescriber.

By definition, the likelihood of a patient receiving a GC prescription during follow-up was greater if they were seen by a ‘high’ GC prescriber compared to a ‘low’ GC prescriber (OR 3.10, 95 % CI 2.90–3.31). The probability of receiving a GC prescription increased with each decade of patient age for both ‘low’ (OR 1.15, 95 % CI 1.11–1.20) and ‘high’ (OR 1.26, 95 % CI 1.23–1.29) prescriber groups (Table 4). This effect differed significantly between the two prescriber groups (*p* <0.001), suggesting that although all older patients were more likely to receive GCs, the effect of age was greater in those seen by a ‘high’ prescriber. In other words, high prescribers were even more likely to prescribe GCs in elderly patients.

**Table 4:** Effect of baseline characteristics on GC prescriptions according to prescriber tendency

Variable	Low prescriber group (OR, 95 % CI)	High prescriber group (OR, 95 % CI)	*p* value^**^
Demographics			
Age (decades)	1.15, 1.11–1.20^*^	1.26, 1.23–1.29^*^	<0.001
Gender (female)	0.98, 0.87–1.10	0.89, 0.82–0.97^*^	0.211
Current smoker (versus never)	1.09, 1.02–1.17^*^	1.07, 1.02–1.13^*^	0.744
GC-associated comorbidities			
Osteoporosis	1.49, 1.14–1.95^*^	1.84, 1.50–2.25^*^	0.225
Avascular necrosis	2.28, 0.32–16.22	1.84, 0.36–9.51	0.870
Myopathy	2.29, 0.57–9.15	1.23, 0.45–3.38	0.479
Diabetes mellitus	0.88, 0.72–1.08	1.02, 0.88–1.18	0.257
Cardiovascular disease	1.38, 1.04–1.84^*^	1.62, 1.31–2.00^*^	0.373
Hypertension	1.08, 0.95–1.24	1.29, 1.17–1.41^*^	0.039
Hyperlipidaemia	1.10, 0.92–1.31	1.07, 0.94–1.22	0.829
Peptic ulcer disease	1.36, 1.06–1.74^*^	1.33, 1.09–1.63^*^	0.910
Pancreatitis	1.00, 0.49–2.05	1.44, 0.81–2.54	0.442
Depression	1.09, 0.96–1.23	1.09, 0.99–1.20	0.994
Insomnia	1.27, 1.08–1.50^*^	1.36, 1.20–1.54^*^	0.548
Psychosis	1.14, 0.60–2.17	1.29, 0.78–2.14	0.767
Prior use			
GC prescription prior to follow up	6.52, 5.51–7.71^*^	11.76, 10.24–13.51^*^	<0.001

*GC* glucocorticoid, *OR* odds ratio, *CI* confidence interval

^*^Significant predictors of GC prescriptions (unadjusted)

^**^ *p* value indicates significance of any differing effect between high and low prescriber groups

It was hypothesised that those with a greater tendency to prescribe GCs may be prescribing inappropriately to those with baseline GC-associated comorbidities. This was only seen for prescribers whose patients had a baseline diagnosis of HTN, who were more likely to receive a GC prescription in the ’high’ prescriber group (OR 1.29 95 % CI 1.17–1.42), but not in the ‘low’ prescriber group (OR 1.08 95 % CI 0.95–1.24), with the effect differing significantly between groups (*p* = 0.039).

## Discussion

This study set out to describe the utilisation of GC therapy for RA in primary care, patterns of GC prescribing and to examine the influence of patient factors and prescriber tendency on GC prescribing amongst GPs for patients with RA. Nearly half the cohort received a GC prescription in primary care during follow-up, consistent with the findings of the QUEST-RA study [23]. The population distribution of the mean prednisolone-equivalent daily dose was 7.5 mg, taken for around 25 % of follow-up time in GC users. This average dose of 7.5 mg is within the European League Against Rheumatism (EULAR) definition of low-dose therapy of ≤7.5 mg per day [24], and in keeping with studies reporting efficacy [25–28] and reduced adverse effects with low-dose therapy[4]. However, more than 50 % were prescribed doses >10 mg per day at some point and nearly 20 % were prescribed doses greater than 30 mg per day. The indication for prescriptions is not available in CPRD, therefore it is difficult to know whether high-dose steroids were prescribed for the patient’s RA or for other indications. The median cumulative duration of time spent taking GCs was 0.8 years/10 months with the interquartile range spanning from 0.15 years/2 months to 2.56 years. This highlights that some patients are taking GCs for longer than recommended [29], placing them at increased risk of developing SEs [30, 31].

As expected, the presence of certain inflammatory comorbidities at diagnosis, in particular inflammatory lung conditions, influenced GC prescribing. Of concern, GC therapy was prescribed more commonly in certain higher risk populations, including older patients and those with CVD. These findings are in keeping with a recent prospective RA study that found rheumatologists were more likely to prescribe GCs and less likely to commence DMARDs in patients who develop RA at an older age, who were also more likely to have comorbidities including CVD, HTN and DM [32]. The authors postulated that this might be due to clinician concerns about the potential side effects of DMARDs in older patients, particularly those with more comorbidities. However, they also point out that DMARDs are well tolerated in older patients [33] compared to the potential difficulties of GC SEs in older patients. It has been shown that RA patients taking ≥7.5 mg prednisolone per day are at increased risk of CVD [34, 35]. Pre-existing CVD, HTN and smoking, an important risk factor for CVD, may worsen cardiovascular outcomes further. Although patients with baseline DM and hyperlipidaemia received fewer GC prescriptions, it was also concerning that other baseline conditions such as PUD had no influence on GC prescribing.

Baseline use of methotrexate and sulfasalazine was associated with less GC prescribing and is in keeping with the knowledge that early treatment of RA within the ‘window of opportunity’ leads to lower disease activity [36], and potentially reduced GC requirement. Several studies have suggested that GC prescribing is also influenced by biological DMARDs (bDMARDs), which have been shown to have a GC-sparing effect [37–40]. In the UK, bDMARDS can only be prescribed by rheumatologists and this data is therefore not captured by CPRD. Therefore this study was unable to assess the influence of these agents on GC prescribing.

The strengths of this study include the large cohort size and the real-life setting in which CPRD data is captured. All oral GC prescriptions were recorded within the database, meaning there was no missing prescription data. The main limitation of this study design was the lack of access to measures of disease activity, which would be expected to be important in understanding which patients receive GC prescriptions. The information needed for standard measures of disease activity such as EULAR response and disease activity score (28-joint count) (DAS28) are not routinely collected by GPs and were therefore not available on the CPRD database. A second limitation of this study is that it was unable to assess the influence of rheumatologist prescribing practices or advice on GC prescribing in primary care as rheumatologist prescribing data is not captured in CPRD. However, it is likely that some GC prescriptions will be initiated by a rheumatologist and continued in primary care. On other occasions, GPs may initiate GCs knowing that this is the practice/recommendation of the treating rheumatologist when faced with active disease. This interaction between prescribers is of interest and warrants further investigation in the future.

## Conclusions

In conclusion, this study has found that 50 % of patients with incident RA were prescribed GCs in UK primary care for 25 % of the time they were observed. Of those who received GCs, more than 50 % were prescribed doses >10 mg per day and nearly 20 % were prescribed doses greater than 30 mg per day. Many GPs and rheumatologists may be surprised by the proportion of patients, the dosages prescribed and the duration of use of GCs in primary care, highlighting the need to be aware of GC use in this setting in order to avoid excess exposure and associated side effects. Certain baseline comorbidities influenced GC prescribing, including some high-risk patient groups that were more likely to receive GC prescriptions. This information is useful for both rheumatologists and GPs involved in the care of patients with RA because it highlights the extent of GC prescribing in primary care and identifies at risk groups more likely to receive GCs. Given the variety of treatment options available for RA, it is important to consider the individual patient’s specific comorbidities and risk of developing GC SEs and introduce alternative therapies where appropriate.

## Abbreviations

bDMARDs: biological disease-modifying anti-rheumatic drugs
CI: confidence interval
CPRD: Clinical Practice Research Datalink
CVD: cardiovascular diseases
DAS28: disease activity score (28-joint count)
DM: diabetes mellitus
DMARDs: disease-modifying anti-rheumatic drugs
EULAR: European League Against Rheumatism
GC: glucocorticoid
GP: general practitioner
HTN: hypertension
IQR: interquartile range
NSAIDs: non-steroidal anti-inflammatory drugs
OR: odds ratio
PUD: peptic ulcer disease
RA: rheumatoid arthritis
SE: side effect
UK: United Kingdom
